# Modulating the gut microbiota to enhance immune checkpoint inhibitor efficacy in colorectal cancer: mechanisms, therapeutic strategies, and clinical perspectives

**DOI:** 10.1080/19490976.2026.2652476

**Published:** 2026-04-14

**Authors:** Yan Chen, Yi-Tong Wang, Jing-Yi Hu, Er-Cong Wang, Xia-He Jia, Jun Wang, Guang-Tao Min, Wei-Lin Jin, Lei Jiang

**Affiliations:** a Department of Stomatology, The First Hospital of Lanzhou University, Lanzhou, People's Republic of China; b School of Basic Medical Sciences, Lanzhou University, Lanzhou, People's Republic of China; c The First School of Clinical Medicine, Lanzhou University, Lanzhou, People's Republic of China; d Department of General Surgery, The First Hospital of Lanzhou University, Lanzhou, People's Republic of China; e Institute of Cancer Neuroscience, Medical Frontier Innovation Research Center, The First Hospital of Lanzhou University, Lanzhou, People's Republic of China

**Keywords:** Immune checkpoint inhibitors, gut microbiota, colorectal cancer, microbiome-targeted therapy, circadian rhythm

## Abstract

Immune checkpoint inhibitors (ICIs) have revolutionized cancer treatment, yet their efficacy in colorectal cancer (CRC) remains limited to a minority of patients with microsatellite instability-high (MSI-H) tumors, leaving the majority with microsatellite stable (MSS) disease unresponsive. The gut microbiota, a key regulator of host immunity, has emerged as a pivotal determinant of ICI response. This review delineates the dual role of the gut microbiome—encompassing specific bacterial strains, their metabolites, and bioactive components such as extracellular vesicles (EVs) and outer membrane vesicles (OMVs)—in either enhancing or impairing ICI efficacy through complex interactions with the host immune system. We further explore the emerging concept of gut microbiota circadian rhythms and their potential to inform personalized chrono-immunotherapy paradigms. Furthermore, we synthesize promising microbiota—targeting strategies as adjunctive approaches to overcome resistance and augment ICI efficacy in CRC. Finally, we present selected clinical evidence and outline future perspectives to expand the clinical benefit of immunotherapy in CRC patients.

## 1. Introduction

Colorectal cancer (CRC) remains a leading cause of global cancer morbidity and mortality, representing a significant clinical and public health burden. In 2020, there were over 1.9 million new CRC cases and 935,000 deaths worldwide, with incidence rates projected to rise further by 2040.^1^ While early-stage CRC is often curable with surgery, advanced or metastatic disease presents a formidable therapeutic challenge, with traditional chemotherapy and targeted therapy offering limited long-term survival benefits for many patients.^2^

In recent years, the advent of immunotherapies such as immune checkpoint inhibitors (ICIs) has brought revolutionary breakthroughs to the treatment of malignant tumors.^3^ By releasing the suppression of immune effector cells such as T cells exerted by immune checkpoint molecules, it reactivates the body's anti-tumor immune response.^4^ It has demonstrated significant therapeutic efficacy in various solid tumors, including melanoma,^5-8^ non-small cell lung cancer,^9-11^ and urothelial carcinoma.^12-14^ In the field of CRC treatment, ICIs have also become a research hotspot, but they still face numerous challenges in clinical application.

The efficacy of ICIs is strikingly heterogeneous and is largely contingent upon the tumor's molecular phenotype. Tumors exhibiting high microsatellite instability (MSI-H) or mismatch repair deficiency (dMMR), which account for approximately 15% of CRC, display a hypermutated genotype, abundant tumor-infiltrating lymphocytes, and consequently, exhibit remarkable sensitivity to PD-1 blockade.^15^^,^^16^ However, the majority of CRC patients are classified as proficient mismatch repair (pMMR) or microsatellite stable (MSS) type, and this group generally exhibits a low response rate to monotherapy with ICIs and constitutes the primary source of primary resistance.^17^ On the one hand, pMMR/MSS tumors typically exhibit a low tumor mutation burden (TMB), yielding a limited number of neoantigens that struggle to sufficiently activate the anti-tumor immune response.^18-20^ On the other hand, the tumor microenvironment (TME) harbors a substantial accumulation of immunosuppressive cells and molecules, creating a state of profound immune suppression that impedes the therapeutic efficacy of ICIs.^21^ Additionally, intrinsic oncogenic pathways within tumors can actively exclude T cells from the tumor core.^22^ For example, activation of the Wnt/β-catenin signaling pathway has been shown to suppress the recruitment of dendritic cells (DCs) and effector T cells by reducing the expression of T cell-recruiting chemokines such as CXCL10, thereby leading to immune rejection and resistance to ICIs.^23^ This cellular autonomy mechanism adds another layer of complexity to the immunosuppressive landscape of MSS CRC. Consequently, pMMR/MSS tumors are termed “cold tumors”, and the inherent resistance mechanisms of these “cold tumors” pose significant challenges to immunotherapy for CRC. Identifying strategies to convert these “immunologically cold” MSS tumors into “hot” tumors responsive to immunotherapy is a paramount focus of current research.

The gut microbiota—recognized as the human body's “second genome”—has garnered significant attention for its regulatory role in numerous diseases. Given the direct association between the site of CRC development and the gut microbiota, multiple studies have demonstrated that the gut microbiota can modulate the progression of CRC. Certain bacterial strains have been demonstrated to be enriched in CRC patients, including polyketide synthase-positive *Escherichia coli* (pks^+^*E. coli*),^24^^,^^25^ enterotoxigenic *Bacteroides fragilis* (ETBF),^26^^,^^27^ *Enterococcus faecalis* (*E. faecalis*),^28^ and *Fusobacterium nucleatum* (*F. nucleatum*).^29-32^ These pathogenic bacteria promote the progression of CRC through mechanisms such as DNA damage, inflammation induction, disruption of the intestinal barrier, and immunosuppression. In contrast, the antitumor properties of certain probiotics are becoming increasingly evident. Among these, the most widely used are *Bifidobacterium*^33-35^ and *Lactobacillus,*^36-38^ which exert their antitumor effects primarily by activating antitumor immunity, protecting the intestinal barrier, and regulating metabolic pathways. Moreover, the gut microbiota has been demonstrated to be highly correlated with the therapeutic efficacy of ICIs.^39^ Its mechanism of action is complex and multidimensional, exerting a dual influence on the therapeutic efficacy of ICIs through multiple pathways.

This review aims to elucidate the potential mechanisms underlying the dual effects of the gut microbiota on ICI therapy for CRC. This review further explores the potential indirect influence of circadian rhythms on immunotherapy efficacy and targeted strategies to enhance treatment outcomes by modulating the microbiome. It also presents early clinical studies on combined therapies involving microbiota and ICIs, offering clinical perspectives. This research provides novel insights and targets for optimizing ICI treatment regimens and enhancing the benefits of immunotherapy for CRC patients.

## 2. Mechanisms of gut microbiota in modulating ICI response in CRC

The efficacy of ICIs in cancer treatment fundamentally depends on the patient's inherent antitumor immune response, which is normally suppressed. The gut microbiota profoundly shapes this immunological landscape through multiple mechanisms. A substantial body of evidence, primarily derived from preclinical mouse models, has begun to elucidate the causal mechanisms by which specific gut microbes modulate ICI efficacy. Where possible, we also highlight correlative findings from human cohorts that support the clinical relevance of these pathways.

### 2.1. Specific microbe-mediated immune activation

**Figure 1.**
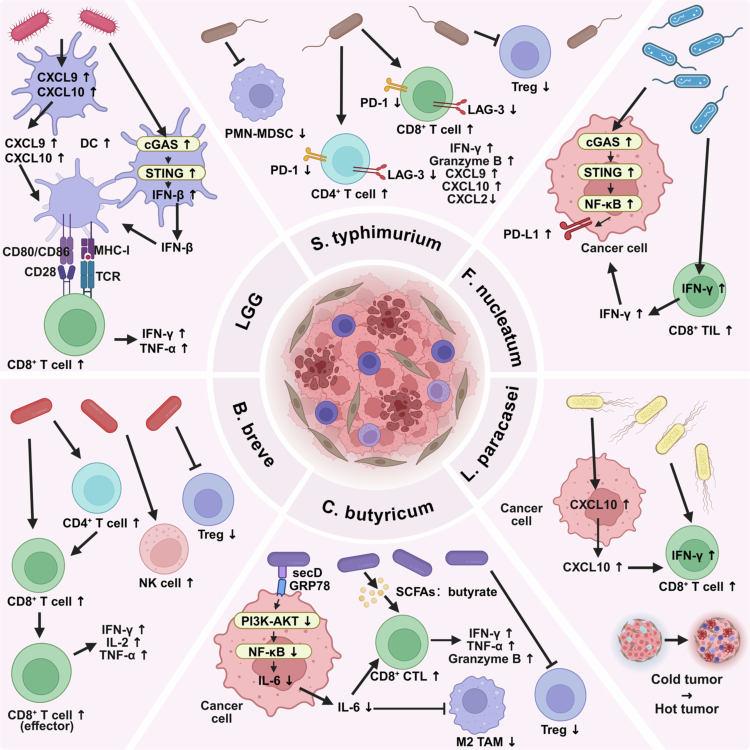
Specific gut bacterial strains modulate the tumor immune microenvironment to enhance ICI efficacy. Multiple probiotic and commensal bacteria promote antitumor immunity through distinct mechanisms, collectively contributing to the conversion of immunologically “cold” tumors into “hot” tumors responsive to ICI therapy. LGG activates the cGAS/STING signaling pathway in DCs, inducing IFN-*β* production and promoting DC maturation, which enhances antigen presentation and T-cell activation. *B. breve*JCM92 increases systemic and intratumoral CD8⁺ T cells, effector CD8⁺ T cells, and NK cells while reducing Treg proportions, thereby boosting cytotoxic activity. *C. butyricum* acts through two complementary mechanisms: (1) its surface protein secD binds to the GRP78 receptor on CRC cells, inhibiting PI3K-AKT-NF-κB signaling and reducing IL-6 secretion, thereby relieving IL-6-mediated suppression of CD8⁺ T cells; and (2) its metabolite butyrate directly activates CD8⁺ T cell cytotoxicity by upregulating IFN-*γ*, TNF-*α*, and granzyme B. *L. paracasei* SH2020 induces tumor cells to secrete CXCL10, a chemokine that recruits CD8⁺ T cells into the tumor microenvironment and upregulates their IFN-*γ* expression. *F. nucleatum* activates the STING/NF-κB pathway to upregulate PD-L1 expression on tumor cells while concurrently increasing infiltration of IFN-γ⁺ CD8⁺ TILs, thereby enhancing sensitivity to PD-L1 blockade in specific contexts. *S. typhimurium* (attenuated strain) reduces the expression of inhibitory checkpoint molecules (PD-1, LAG-3) on T cells, alleviating T-cell exhaustion and improving ICI efficacy. This figure was created using BioRender (https://biorender.com/).

A favorable gut microbiome can orchestrate a proinflammatory, antitumor immune milieu conducive to ICI action (Figure 1).

#### 2.1.1. Enhancement of antigen presentation and DCs function

Certain gut commensals can directly modulate DCs function, a pivotal step in initiating adaptive immunity. A preclinical study using murine CRC models demonstrated that *Lactobacillus intestinalis* (*L. intestinalis*) facilitates the recruitment and activation of DCs into the TME by promoting tumor cell-derived CCL5 release, suggesting a potential mechanism for its antitumor effects.^40^ Mechanistic studies using murine tumor models and bone marrow-derived dendritic cells (BMDCs) have demonstrated that *Lactobacillus rhamnosus* GG (LGG) activates the intracellular cGAS/STING signaling pathway, leading to the production of type I interferon (e.g., IFN-*β*), which in turn promotes the activation and maturation of DCs and enhances the efficacy of anti-PD-1 therapy.^41^ Activated DCs more efficiently present tumor antigens to T cells, a critical first step for ICI-mediated reinvigoration.

#### 2.1.2. Promotion of cytotoxic CD8^+^ T cell (CD8^+^ CTLs) infiltration and function

The presence and functionality of tumor-infiltrating CD8^+^ T lymphocytes (CD8^+^ TILs) are strong predictors of ICI response. Beneficial bacteria directly enhance this axis. Preclinical studies in MC38 colon carcinoma-bearing mice have demonstrated that oral administration of *Bifidobacterium breve* (*B. breve*) JCM92 enhances antitumor immunity by increasing the frequencies of systemic and intratumoral CD8⁺ T cells, effector CD8⁺ T cells (CD8⁺ Teff), and natural killer cells (NK cells), while reducing regulatory T cell populations. Notably, this immune-stimulating effect was strain-specific, as another *B. breve* strain (Bb03) exhibited antitumor activity alone but failed to boost the efficacy of oxaliplatin or PD-1 blockade.^42^ Moreover, *Lactobacillus paracasei* (*L. paracasei*) SH2020 has been shown to enhance anti-PD-1 efficacy by promoting CD8⁺ T-cell infiltration into tumors. Mechanistically, this strain induces tumor cells to secrete CXCL10, a chemokine critical for T-cell recruitment, as demonstrated by in vitro stimulation assays and in vivo CXCL10 neutralization experiments that abolished its antitumor effects.^43^ In addition, *Clostridium butyricum* (*C. butyricum*) has been shown to enhance anti-PD-1 efficacy in CRC through two complementary mechanisms. First, its surface protein secD binds to the GRP78 receptor on CRC cells, inhibiting PI3K-AKT-NF-κB signaling and reducing IL-6 secretion, thereby relieving IL-6-mediated suppression of CD8⁺ T cells—a mechanism validated by GRP78 knockout and IL-6 neutralization experiments. Second, its metabolite butyrate directly activates CD8⁺ T cell cytotoxicity.^44^ Notably, in murine models, *F. nucleatum* activates the STING/NF-κB signaling pathway to upregulate PD-L1 expression on tumor cells while increasing the infiltration of IFN-γ^+^ CD8⁺ TILs, thereby enhancing sensitivity to PD-L1 blockade. Clinical cohort analyzes further revealed that the presence of *F. nucleatum* in tumor tissues correlates with improved therapeutic responses to PD-1 blockade in patients with CRC.^45^

#### 2.1.3. Modulation of immunosuppressive cells

Gut microbiota also reshape the tumor immune microenvironment by modulating immunosuppressive cell populations. A key mechanism of action is the restraint of regulatory T cells (Tregs). Studies in murine CRC models have demonstrated that oral administration of *B. breve* JCM92 reduces the proportion of Tregs among immune cells, thereby weakening their suppression of effector T cells.^42^ Similarly, *C. butyricum* inhibits the polarization of M2-type tumor-associated macrophages (M2 TAMs) and decreases Treg accumulation, collectively alleviating local immunosuppression in tumors.^44^ Furthermore, attenuated bacteria such as Attenuated *Salmonella typhimurium* (*S. typhimurium*) can reduce the expression of inhibitory checkpoint molecules like PD-1 and LAG-3 on T cells, alleviating T-cell exhaustion and further improving the efficacy of ICIs.^46^

Although the aforementioned studies highlight the immunomodulatory effects of individual bacterial strains, it is important to recognize that in clinical settings, the gut microbiota constitutes a complex ecological community. The ultimate impact on host immunity and ICI efficacy depends not only on the presence or absence of specific strains but also on the dynamic interactions among community members, including competition, cross-feeding, and metabolic cooperation. The key point is that these ecological interactions often converge on the production of metabolites—the primary messengers that transmit microbial signals to the host immune system. For example, the production of the immunomodulatory metabolite butyrate, which will be discussed in the next section, often relies on cross-feeding between multiple bacterial species.^47^ Therefore, while individual strains offer functional potential, it is ultimately the community-derived pool of metabolites that coordinates the immune response. This perspective naturally leads us to explore the specific role of microbial metabolites in shaping the efficacy of ICI therapy.

### 2.2. Microbial metabolites as immunomodulatory messengers

**Figure 2.**
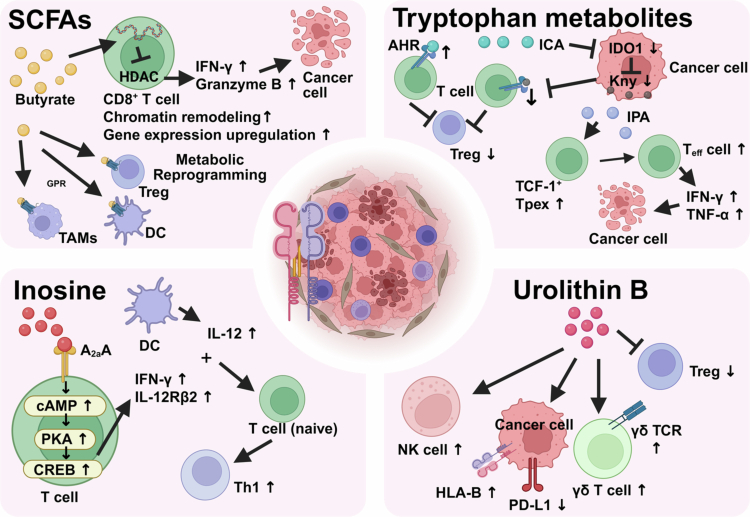
Microbial metabolites activate antitumor immunity. Multiple microbial metabolites exert immunomodulatory effects through distinct signaling pathways, collectively shaping the tumor immune microenvironment and influencing responses to ICI therapy. SCFAs. Butyrate, a principal SCFA, inhibits HDACs, leading to chromatin remodeling and upregulation of gene expression in CD8⁺ T cells. This enhances their effector functions, including increased production of IFN-*γ* and granzyme B, thereby promoting cytotoxic T-cell-mediated tumor cell killing. SCFAs also modulate the function of TAMs, DCs, and Tregs through metabolic reprogramming and GPR activation. Tryptophan metabolites. IPA and ICA modulate the IDO1/Kyn/AHR axis. ICA inhibits IDO1 expression in tumor cells, reducing Kyn production. Both IPA and ICA compete with Kyn for binding to the AHR on T cells, with ICA exhibiting higher affinity for AHR. This competition inhibits Treg differentiation and promotes the expansion of Tpex, characterized by Tcf-1 expression, which can differentiate into effector CD8⁺ T cells producing IFN-*γ* and TNF-*α*. Inosine. Produced by *B. pseudolongum* and *A. muciniphila*, inosine activates the A₂AR on T cell surfaces. This triggers the cAMP-PKA-CREB signaling cascade, leading to upregulation of IL-12Rβ2 on T cells. In the presence of IL-12 from DCs, this pathway promotes the differentiation of naive CD4⁺ T cells into IFN-*γ*-producing Th1 cells, which subsequently support CD8⁺ cytotoxic T lymphocyte activation and enhance antitumor immunity. UB. This gut microbiota-derived metabolite exerts pleiotropic immunomodulatory effects. UB enhances gut microbiota diversity, increases the number of NK cells and γδ T cells, upregulates γδ T cell receptor (γδ TCR) expression, suppresses Tregs activity, downregulates PD-L1 expression, and upregulates HLA-B on tumor cells. These combined actions establish a highly immunoreactive TME that potentiates the efficacy of subsequent immunotherapy. This figure was created using BioRender (https://biorender.com/).

Bacteria-derived small molecules are potent systemic regulators of host immunity, serving as key intermediaries in the microbiota-ICI axis (Figure 2).

#### 2.2.1. Short-chain fatty acids (SCFAs)

SCFAs, primarily acetate, propionate, and butyrate, are key microbial metabolites derived from dietary fiber fermentation in the gut. Accumulating evidence highlights their crucial role in modulating the tumor immune microenvironment and enhancing the efficacy of ICIs.^48^^,^^49^ In murine CRC models, butyrate has demonstrated remarkable immunomodulatory activity. Butyrate produced by *Roseburia intestinalis* (*R. intestinalis*) directly activates CD8^+^ CTLs, enhances their tumor-killing capacity, and significantly improves response rates to anti-PD-1 therapy.^50^ Mechanistically, butyrate acts as an inhibitor of histone deacetylases (HDACs), leading to chromatin remodeling that promotes the expression of effector molecules (e.g., IFN-*γ*, granzyme B) in CD8^+^ T cells and sustains their stem-like properties and long-term immunological memory.^51^ Furthermore, SCFAs can indirectly modulate the functions of DCs, Tregs, and macrophages through activation of G-protein-coupled receptors (e.g., GPR41, GPR43) and influencing cellular metabolic reprogramming, collectively shaping an immune-favorable tumor microenvironment.^52^ Notably, the immunoregulatory effects of SCFAs exhibit circadian rhythm and tissue specificity, suggesting their potential for chrono-immunotherapeutic applications.^53^ In summary, SCFAs, particularly butyrate, serve as critical mediators of microbiota-host immune crosstalk and hold promising potential for improving immunotherapy response and overcoming resistance.

#### 2.2.2. Tryptophan metabolites

Tryptophan metabolism is profoundly influenced by the gut microbiota, with indoles and their derivatives representing the primary metabolic products generated through microbial catabolism of tryptophan. Mechanistic studies have revealed that *Lactobacillus gallinarum* (*L. gallinarum*) and its metabolite indole-3-carboxylic acid (ICA) enhance anti-PD-1 efficacy in CRC through modulation of the IDO1/Kyn/AHR axis. ICA inhibits the expression of indoleamine 2,3-dioxygenase 1 (IDO1) in tumors, thereby reducing the production of kynurenine (Kyn). ICA competes with Kyn for binding to the aryl hydrocarbon receptor (AHR) on T cells, exhibiting higher affinity for AHR than Kyn, thereby inhibiting Treg differentiation.^54^ *Lactobacillus johnsonii* (*L. johnsonii*) and *Clostridium sporogenes* (*C. sporogenes*) synergistically produce indole-3-propionic acid (IPA). IPA significantly increases the number of progenitor exhausted CD8^+^ T Cells (Tpex) and CD8⁺ Teff cells. Tpex can differentiate into Teff cells through clonal expansion, secreting cytokines such as IFN-*γ* and tumor necrosis factor-*α* (TNF-*α*) to directly kill tumor cells and enhance the efficacy of immunotherapy.^55^

#### 2.2.3. Inosine

Mechanistic studies using germ-free mice and adoptive T cell transfer models have demonstrated that inosine, a metabolite produced by *Bifidobacterium pseudolongum* (*B. pseudolongum*), enhances ICI efficacy through the adenosine A2A receptor (A_2a_R) on T cells.^56^ Inosine under the combined effects of ICI therapy (e.g., anti-cytotoxic T-lymphocyte-associated protein 4, CTLA-4) and co-stimulatory signals (e.g., IL-12) activates downstream signaling pathways via the A_2a_R on T-cell surfaces. This promotes the differentiation of naive CD4⁺ T cells into T helper 1 cells (Th1 cells) and enhances their functional capabilities (e.g., IFN-*γ* secretion). These activated Th1 cells further activate cells such as CD8⁺ CTLs, collectively enhancing the antitumor immune response and thereby improving the therapeutic efficacy of ICIs. The study further demonstrated that *Akkermansia muciniphila* (*A. muciniphila*) is capable of producing inosine, and its mechanism for enhancing ICIs similarly relies on the A_2a_R signaling pathway. Therefore, inosine supplementation may serve as an adjunctive therapy to ICIs, potentially enhancing patient response rates. Moreover, A_2a_R antagonists should be administered with caution in clinical trials to avoid interfering with the beneficial immunomodulatory of inosine.^56^^,^^57^

#### 2.2.4. Urolithin B (UB)

Studies in murine CRC models have shown that UB, a gut microbiota-derived metabolite, enhances antitumor immune responses through multiple mechanisms. UB treatment increases gut microbiota diversity. It enhances antitumor immune responses by increasing the number of NK cells and γδ T cells, suppressing the activity of Tregs, downregulating PD-L1 expression, and upregulating human leukocyte antigen-B (HLA-B) and γδ T cell receptor expression. The combination of UB and immunotherapy effectively suppresses CRC growth and establishes a highly immunoreactive microenvironment, offering enhanced potential for subsequent immunotherapeutic interventions in CRC patients.^58^

#### 2.2.5. Context-dependent effects of microbial metabolites

**Figure 3.**
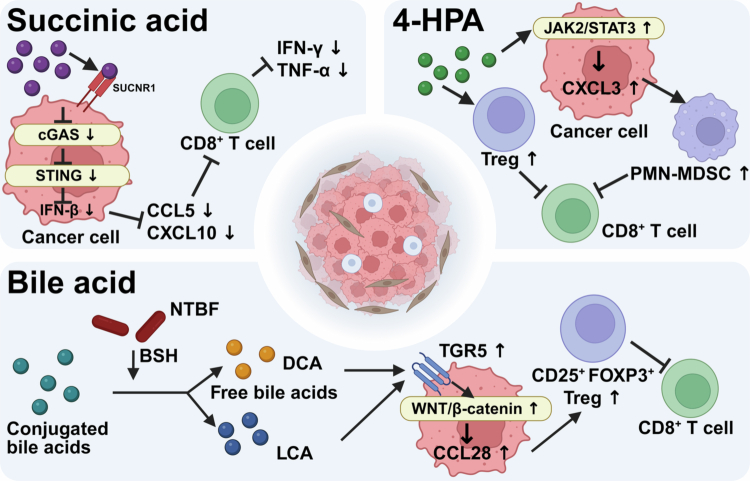
Microbial metabolites suppress antitumor immunity through distinct signaling pathways. Certain gut microbiota-derived metabolites promote an immunosuppressive TME, compromising the efficacy of ICI therapy. This figure illustrates the mechanisms of three key immunosuppressive metabolites discussed in Section 2.2.5. Succinic acid. Derived from *F. nucleatum*, succinic acid activates SUCNR1 on CRC cells, initiating the SUCNR1-HIF-1α-EZH2 signaling axis. This cascade suppresses the cGAS-STING-IFN-*β* pathway, leading to reduced production of Th1-type chemokines CCL5 and CXCL10. Consequently, CD8⁺ T cell trafficking into the TME is impaired, and their cytotoxic function is diminished, contributing to resistance to PD-1 inhibitors. 4-HPA. Produced by *F. nucleatum* and pks⁺ *E. coli*, 4-HPA activates the JAK2/STAT3 signaling pathway in cancer cells, leading to transcriptional upregulation of CXCL3. Elevated CXCL3 promotes the infiltration of PMN-MDSCs into the TME. These PMN-MDSCs suppress CD8⁺ T cell function, thereby dampening antitumor immune responses and compromising the efficacy of PD-1 blockade therapy. Bile acids. Specific genotypes of NTBF that highly express BSH convert conjugated bile acids into unconjugated forms, including DCA and LCA. These free bile acids activate the G-protein-coupled receptor TGR5 and upregulate WNT/β-catenin signaling in tumor cells. This activation promotes the recruitment of immunosuppressive CD25⁺ FOXP3⁺ Tregs into the TME, contributing to the formation of an immunosuppressive niche that may compromise ICI efficacy This figure was created using BioRender (https://biorender.com/).

It is noteworthy that the impact of microbial metabolites on immunotherapy is not always positive; certain metabolites can drive immunosuppression and lead to treatment resistance (Figure 3). Metabolites from *F. nucleatum* exert opposing effects on immunotherapy outcomes in patients with CRC. Its metabolite succinic acid activates succinate receptor 1 (SUCNR1) on CRC cells, thereby initiating the SUCNR1-HIF-1-EZH2 signaling axis and subsequently inhibiting the cGAS-IFN-*β* pathway. This leads to a reduction in Th1-type chemokines (e.g., CXCL10) within the tumor, impeding the migration of CD8^+^ T cells into the TME while simultaneously diminishing their cytotoxicity, resulting in CRC developing resistance to PD-1 inhibitors.^59^ 4-Hydroxybenzeneacetic acid (4-HPA), a metabolite produced by *F. nucleatum* and pks⁺ *E. coli*, enhances CXC motif chemokine ligand 3 (CXCL3) transcription, activates the JAK2/STAT3 signaling pathway, and promotes the infiltration of polymorphonuclear myeloid-derived suppressor cells (PMN-MDSCs) into the TME. As a result, the antitumor response is suppressed, the efficacy of PD-1 antibodies is compromised, and CRC progresses more rapidly in vivo. In contrast, chlorogenic acid can reduce 4-HPA production, alleviate immune suppression associated with gut microbiota dysbiosis, decrease tumor burden, and thereby enhance the therapeutic effect of immunotherapy.^60^ In addition to *F. nucleatum* and pks⁺ *E. coli*, other bacteria enriched in CRC have also been found to participate in immune suppression through metabolite-mediated mechanisms. For instance, specific genotypes of nonenterotoxigenic *Bacteroides fragilis* (NTBF) that highly express bile salt hydrolase (BSH) promote colorectal carcinogenesis by altering the bile acid pool. BSH converts conjugated bile acids into unconjugated forms such as deoxycholic acid (DCA) and lithocholic acid (LCA), which subsequently activate the G-protein-coupled bile acid receptor, leading to upregulation of the *β*-catenin/CCL28 axis in tumor cells. This cascade results in increased intratumoral recruitment of immunosuppressive CD25⁺ FOXP3⁺ Tregs, thereby creating an immunosuppressive microenvironment that may compromise ICI efficacy.^61^

The seemingly contradictory roles of *F. nucleatum* in modulating ICI efficacy highlight the complexity of host‒microbe interactions in CRC. This duality likely reflects context-dependent mechanisms primarily determined by the route of bacterial administration and the resulting mode of action. Direct intratumoral injection of live *F. nucleatum* enables bacterial contact with tumor cells, thereby sensitizing tumors to PD-L1 blockade.^45^ In contrast, oral administration of *F. nucleatum* does not lead to bacterial accumulation in tumors but rather exerts systemic immunosuppressive effects through its metabolites, such as succinic acid^59^ and 4-HPA.^60^ These findings underscore the need for future studies to carefully consider these context-dependent factors, particularly the route of administration and the distinction between local bacterial presence and systemic metabolite effects, when evaluating the potential of targeting *F. nucleatum* for CRC immunotherapy.

### 2.3. Microbial active components

**Figure 4.**
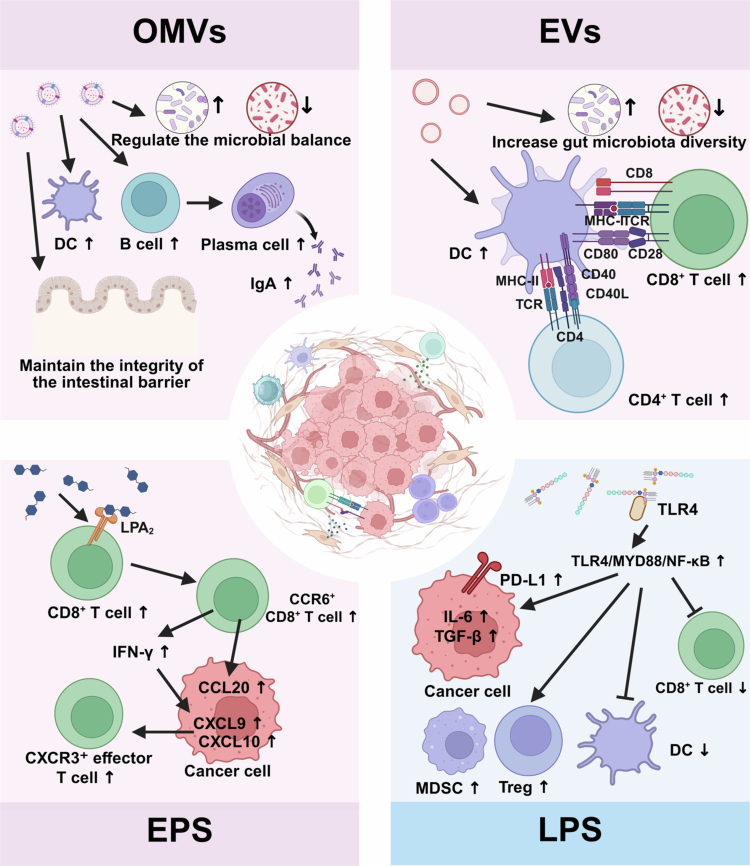
Modulation of ICI efficacy by gut microbiota-derived bioactive components. Beyond live bacteria and their metabolites, specific structural components and secreted factors derived from the gut microbiota function as bioactive carriers that modulate host immunity and influence immune checkpoint inhibitor (ICI) efficacy. OMVs. Derived from *A. muciniphila*, OMVs selectively promote the growth of beneficial bacteria, restoring gut microbiota equilibrium. OMVs activate B cells and DCs, inducing mucosal IgA responses. They also upregulate tight junction proteins to preserve intestinal barrier integrity, thereby maintaining gut homeostasis, alleviating colitis, and enhancing the therapeutic efficacy of immunotherapy against colorectal cancer. EVs. Produced by LGG, LGG-EVs modulate intestinal immunity by enhancing gut microbiota diversity. They increase the proportions of MHC II⁺ DCs, CD4⁺ T cells, and CD8⁺ T cells in tumor tissues, promoting antigen presentation and T cell activation through costimulatory molecules (CD80/CD86 on DCs interacting with CD28 on T cells, and CD40 on DCs engaging CD40L on T cells). Orally administered LGG-EVs synergistically enhance the efficacy of anti-PD-1 therapy in CRC. EPS. EPS induces the generation of CCR6⁺ CD8⁺ T cells by binding to the LPA₂ on CD8⁺ T cells. These cells specifically target CCL20-expressing tumor tissues (e.g., CRC), where they remodel the TME, recruit CXCR3⁺ effector T cells, and synergize with ICIs to enrich tumor-specific cytotoxic T lymphocytes while reducing T cell exhaustion, ultimately enhancing antitumor efficacy. This effect is dependent on tumoral CCL20 expression. LPS. LPS binds to TLR4 on tumor cells, activating the TLR4/MYD88/NF-κB signaling pathway. Persistent activation promotes secretion of immunosuppressive factors (IL-6, TGF-*β*) while concurrently upregulating PD-L1 expression on tumor cell surfaces, enhancing tumor cells' inhibitory effect on T cells. TLR4 signaling also facilitates the recruitment and activation of MDSCs and promotes Treg accumulation, reinforcing the immunosuppressive microenvironment. This figure was created using BioRender (https://biorender.com/).

In addition to intact live bacteria and their classic metabolites, specific structural components and secreted factors derived from the gut microbiota, functioning as highly specialized bioactive carriers, demonstrate significant potential for precisely modulating host immunity, particularly in enhancing the efficacy of cancer immunotherapy (Figure 4).

*A. muciniphila* is capable of releasing bacterial outer membrane vesicles (OMVs). Through membrane fusion, OMVs can selectively promote the growth of beneficial bacteria, thereby restoring equilibrium to the gut microbiota. Additionally, OMVs activate B cells and DCs to induce mucosal IgA responses. OMVs upregulate tight junction proteins to preserve the integrity of the intestinal barrier. Transplantation of OMVs into mouse intestines helps maintains gut homeostasis, alleviates colitis, and enhances the therapeutic efficacy of immunotherapy against CRC.^62^ Studies in murine CRC models have shown that orally administered LGG-derived extracellular vesicles (EVs) modulate intestinal immunity and synergistically enhance anti-PD-1 therapy efficacy. LGG-EV treatment increases the proportions of MHC II⁺ DCs, CD4⁺ T cells, and CD8⁺ T cells within the TME, contributing to improved antitumor immune responses.^63^ Studies have demonstrated that extracellular polysaccharides (EPS) derived from *Lactobacillus bulgaricus* (*L. bulgaricus*) induces the generation of CCR6⁺ CD8⁺ T cells by binding to the LPA₂ receptor on the surface of CD8⁺ T cells. It specifically targets C-C motif chemokine ligand 20 (CCL20) expressing tumor tissues, remodels the TME, recruits CXCR3⁺ Teff, and synergize with ICIs to enrich tumor-specific CTLs while reducing T-cell exhaustion, ultimately enhancing antitumor efficacy. The key prerequisite for EPS to exert its effect is tumors that highly express CCL20 (e.g., CRC). For melanoma that does not express CCL20, EPS is ineffective.^64^ Lipopolysaccharides (LPS), originating from Gram-negative gut bacteria, are found at significantly levels in CRC tissues in situ. LPS binds to toll-like receptor 4 (TLR4) to activate the TLR4/MYD88/NF-κB signaling pathway. Persistent activation of this pathway promotes the secretion of multiple immunosuppressive factors (e.g., IL-6, TGF-*β*) while simultaneously upregulating PD-L1 expression on tumor cell surfaces, thereby enhancing the tumor cells' inhibitory effect on T cells. The activation of TLR4 further promotes the recruitment and activation of MDSCs via the STAT3 signaling pathway, thereby reinforcing the immunosuppressive microenvironment and rendering anti-PD-L1 therapy less effective in overcoming this immunosuppression.^65^

As described in Section 2.1.2, intratumoral *F. nucleatum* activates the STING/NF-κB axis to upregulate PD-L1, a process accompanied by increased infiltration of IFN-γ⁺ CD8⁺ T cells.^45^ This immunostimulatory environment enables PD-L1 upregulation to function as an adaptive resistance mechanism, which paradoxically enhances antitumor efficacy when combined with PD-L1 blockade. In contrast, LPS derived from Gram-negative bacteria activates the TLR4/MYD88/NF-κB pathway, but this pathway is associated with broader immunosuppressive programs, including MDSCs recruitment and persistent inflammation.^65^ Against this backdrop, PD-L1 upregulation occurs within an immunosuppressive microenvironment characterized by a scarcity of effector T cells, thereby limiting the therapeutic benefit of PD-L1 blockade alone. This comparison illustrates that pathogen-associated molecular patterns (PAMPs) can converge on similar immune checkpoints through different mechanisms, yet the net effect on ICI efficacy depends on the broader immunological context—including the balance between effector T cell infiltration and immunosuppressive cell populations, the duration of signaling, and the composition of the tumor microenvironment. Understanding these context-dependent differences is crucial for developing microbiota-targeted strategies that selectively enhance, rather than impair, ICI responses.

## 3. Circadian rhythm and microbiota‒host-immunity crosstalk

Beyond static composition, the temporal dynamics of the gut microbiota, governed by circadian rhythms, represent a crucial and often overlooked dimension that may significantly impact the outcome of ICI therapy.

### 3.1. The association between circadian rhythms and CRC

Circadian rhythms are regulated by the central biological clock in the suprachiasmatic nucleus of the hypothalamus. Key circadian genes include Period (Per1, Per2, Per3), Cryptochrome (Cry1, Cry2), Bmal1, and others.^66^ Circadian rhythm disruption is closely associated with the progression of CRC. Bioinformatics analysis reveals that multiple circadian clock genes (CCGs) are dysregulated and frequently mutated in CRC samples.^67^ The CCG Bmal1 suppresses CRC development by regulating the Hippo signaling pathway in intestinal stem cells. Conversely, Bmal1 deficiency or light disruption enhances the self-renewal capacity of intestinal stem cells, significantly accelerating CRC progression.^68^ Further research has demonstrated that loss of function in the Bmal1 gene accelerates APC heterozygosity loss, thereby triggering abnormal activation of the Wnt signaling pathway and ultimately accelerating the progression of CRC.^69^

### 3.2. Circadian rhythm characteristics and regulatory factors of the gut microbiota

The gut microbiota is not static but exhibits a pronounced circadian rhythm, characterized by regular changes in microbial composition, abundance, and metabolic activity throughout the day‒night cycle.^70-72^ Host CCGs are key drivers of microbial circadian rhythms. Research has revealed that following the knockout of the Bmal1 gene, the circadian rhythm of the gut microbiota in mice was entirely abolished, with the oscillatory relative abundance of the phyla Bacteroidetes and Firmicutes also ceasing to occur. It was also discovered that the circadian rhythms of gut microbiota exhibit gender differences, with female mice displaying more pronounced rhythmicity in their microbial communities than their male counterparts.^73^ Recent research has also corroborated this finding.^74^ Dietary patterns also influence microbial rhythms. Following a high-fat, low-fiber diet, both male and female mice exhibited a decline in the rhythmicity of their microbial communities, with the distribution of peak activity disrupted.^74^ Supplementing exogenous melatonin can repair.^75^ Furthermore, light exposure also influences the circadian oscillations of the gut microbiota.^76^^,^^77^ The circadian rhythms between host and microbiota are not unidirectional but represent a bidirectional interaction. On the one hand, the host provides a stable environment for the microbial community by regulating core circadian genes.^78^ On the other hand, the gut microbiota and its metabolites can exert a bidirectional influence on the host's circadian rhythm by modulating the expression of host circadian genes, thereby forming a host‒microbiota rhythmic interaction network.^53^^,^^79^

### 3.3. The impact of disrupted circadian rhythms on ICI therapy

#### 3.3.1. Circadian disruption of gut microbiota

When the rhythmicity of the gut microbiota is disrupted by external factors, it leads to alterations in microbial composition and abnormal circadian fluctuations in metabolic products.^70^ Research using a mouse model of CRC has revealed that disruptions in the circadian rhythm alter the diversity and abundance of gut microbiota, while cancer progression further exacerbates these changes.^80^ Firstly, the circadian rhythm of the gut microbiota directly influences host immune function by regulating the expression of immune-related genes and the activity of immune cells.^74^ Second, abnormal fluctuations in gut microbial metabolites disrupt the homeostasis of key metabolites such as SCFAs,^81^^,^^82^ bile acids,^83^ and tryptophan and its metabolites.^84^ Research has found that the bile acid metabolic pathway is significantly enriched in the gut microbiota metabolites of circadian rhythm-disrupted mice, with taurocholate (TCA) showing a marked increase across all disrupted models. TCA regulates MDSC function at both the epigenetic and protein levels. TCA promotes the massive accumulation of MDSCs in the TME and activates their immunosuppressive functions, thereby suppressing CD8⁺ T cell function and shaping an immunosuppressive TME.^85^ Therefore, the circadian clock regulates the gut microbiota and its metabolites, playing a crucial role in the evolution of the TME and cancer progression.^86^ Disruption of microbial circadian rhythms may affect the efficacy and safety of ICI therapy.

#### 3.3.2. Intrinsic circadian rhythms in immune cells

Immune cells themselves possess intrinsic circadian rhythms, with oscillating molecular circadian rhythms detected in nearly all cells involved in immune system function.^87-89^ These dynamic changes directly influence the intensity of antitumor immune responses. Research has revealed that DCs and CD8⁺ T cells exhibit circadian rhythmicity in anti-tumor function.antitumor functions. Specifically, the circadian rhythm of DCs governs the rhythmic antitumor activation of CD8⁺ T cells, a response dependent on the circadian expression of the costimulatory molecule CD80. Consequently, immunotherapy synchronized with the rhythms of DCs significantly enhances antitumor efficacy. These circadian rhythmicity findings were demonstrated in mice and suggest similar effects in humans.^90^ Similarly, in CRC animal models, studies have revealed that the abundance of PD-L1^+^ MDSCs is regulated by circadian rhythms, peaking during the early active phase. Administering anti-PD-L1 at this time more effectively blocks the immunosuppressive effects of MDSCs, promotes tumor infiltration by CD8^+^ T cells, and significantly enhances antitumor efficacy. In contrast, administration during the resting phase fails to effectively activate antitumor immunity due to low MDSCs abundance, resulting in significantly reduced therapeutic effects.^91^ Administering ICIs when the immune system is most “alert” can reduce T-cell suppression and trigger stronger, more durable antitumor responses.

Given that both gut microbiota and immune cells exhibit circadian rhythms, do the rhythmic fluctuations of microbial metabolites such as SCFAs directly influence the circadian rhythmicity of tumor-infiltrating CD8⁺ T cells or DCs? Considering that SCFAs undergo circadian oscillations and can modulate T-cell function, their rhythmicity likely participates in the circadian regulation of antitumor immunity. Therefore, theoretically, timing ICI treatment to coincide with appropriate circadian rhythms could enhance the efficacy of immune responses.^92^

### 3.4. Chrono-immunotherapy strategy based on circadian rhythms

Chronotherapy is a promising cancer treatment approach designed to leverage circadian rhythms to enhance therapeutic efficacy and mitigate adverse reactions.^93^ An analysis synthesizing 18 retrospective studies revealed that early-time-of-day (ToD) infusion of ICIs can enhance progression-free or overall survival by up to four times compared to late-ToD dosing across multiple cancer types, including melanoma, non-small cell lung cancer, and renal cell carcinoma.^94^ Therefore, developing time-based medical strategies offers new avenues for optimizing the gut microbiome and regulating the immune system. Tailoring treatment plans to an individual's circadian rhythm characteristics can enhance the efficacy of ICIs while reducing adverse reactions. For example, adopting circadian rhythm-based eating patterns to regulate the circadian rhythms of the microbiome and immune system.^95^ A high-fiber diet during daylight hours helps maintain the rhythmic balance of the gut microbiota, promotes the proliferation of SCFA-producing bacteria, and improves the TIME.^96^ Increase physical exercise to improve gut microbiota enrichment and regulate immunity.^97^ Using wearable biosensors to continuously and remotely monitor phase markers of patients' circadian rhythms, providing more personalized treatment recommendations based on the individual physiological stages associated with immunotherapy.^98^ Based on the host's circadian rhythms, including gut microbiome rhythms and immune system rhythms, the administration timing of ICIs is personalized. Although current research into microbiome-based circadian rhythm strategies for immunotherapy remains in its preliminary stages, existing cross-disciplinary studies have provided both theoretical foundations and practical avenues for exploration. Further studies are required to validate their efficacy and safety.

These findings lay the groundwork for chrono-modulated interventions, which we will explore in the context of broader microbiota-targeting strategies in Section 4.

## 4. Therapeutic strategies to modulate gut microbiota for enhanced ICI efficacy

**Figure 5.**
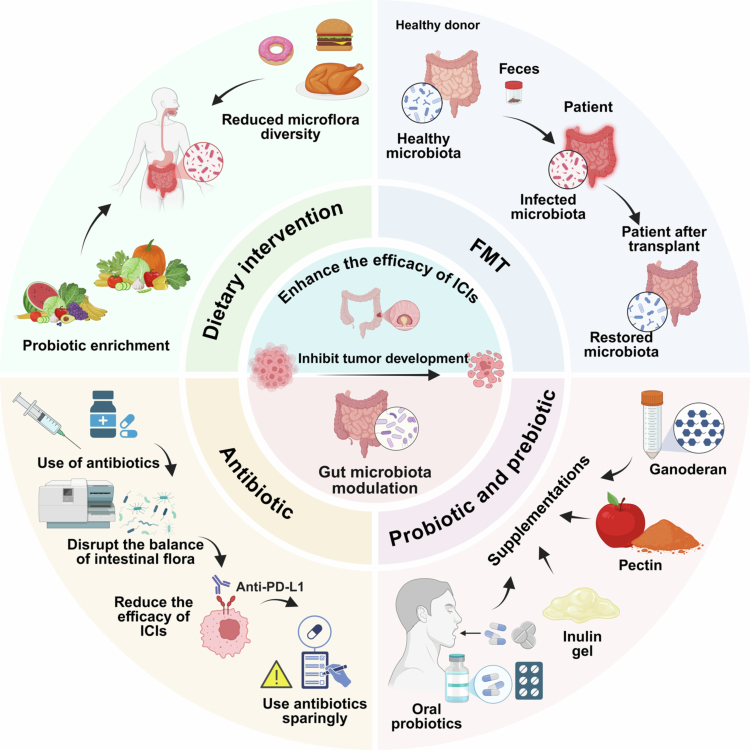
Therapeutic strategies for modulating gut microbiota to enhance ICI efficacy. Dietary intervention. Dietary components, particularly fermentable fibers such as pectin, promote the growth of SCFA-producing bacteria. This increases SCFA production, which has direct immunomodulatory effects, including enhanced CD8⁺ T cell function and improved tumor immune microenvironment. Dietary strategies represent a foundational, low-risk approach to microbiota modulation. FMT. Fecal samples from healthy donors with diverse, balanced microbiota are transplanted into patients with dysbiosis (often following antibiotic pretreatment). This restores gut microbial diversity and composition, leading to probiotic enrichment and inhibition of tumor development. FMT has shown particular promise in restoring ICI responsiveness in refractory patients. Probiotic and prebiotic supplementation. Oral administration of specific probiotics or prebiotics (e.g., pectin, inulin gel) directly enriches beneficial bacterial populations. These interventions enhance antitumor immunity by modulating the gut microbiota composition and metabolic output, thereby improving ICI efficacy. Judicious antibiotic use. Broad-spectrum antibiotics disrupt the balance of intestinal flora, reducing microbial diversity and depleting immunostimulatory commensal bacteria. This dysbiosis is associated with poorer ICI outcomes. Therefore, antibiotics should be used sparingly and only when clinically indicated in patients receiving ICI therapy. This figure was created using BioRender (https://biorender.com/).

Due to its high plasticity, the gut microbiota constitutes a promising target for combination therapeutic strategies, thereby opening new treatment avenues for patients with CRC (Table 1). Interventions including dietary modifications, FMT (Fecal Microbiota Transplantation), targeted probiotic and prebiotic supplementation, and judicious antibiotic use have emerged as promising adjunctive approaches to improve the efficacy of immunotherapy (Figure 5).^99^

**Table 1.** Comparative strategies for modulating gut microbiota to enhance ICI efficacy.

Strategy categories	Principle of operation	Representative material/method	Maturity (clinical)	Key challenges	Clinical prospects
Dietary intervention	Provide substrates to selectively stimulate the growth of beneficial bacteria.	High-fiber diet, Mediterranean diet.	Level 2	Individual responses vary significantly, making precise control difficult.	As a foundational, low-risk lifestyle intervention, it can be easily integrated into clinical guidelines.
FMT	Comprehensively rebuild the intestinal microbiota ecosystem.	Screened stool samples from healthy individuals or respondents.	Level 2	Safety risks (e.g., infection, immune reactions), complex donor screening, difficulty in standardization, regulatory barriers.	For refractory patients, it may serve as a potent means to “reset” the microbiota.
Probiotics and specific prebiotics	Directly supplement beneficial bacteria to colonize and regulate local immunity.	LGG, inulin, pectin.	Level 1–2	High strain specificity, uncertain colonization success rate, and safety require long-term observation.	As a standardized, safe adjunctive supplement with significant potential, large-scale RCTs are required.
Targeted antimicrobial therapy	Selectively eliminate pathogens that promote tumor growth or suppress immunity.	Liposomal antibiotics.	Level 0	Identify genuine pathogenic targets while avoiding disruption of beneficial microbial communities, which may trigger bacterial resistance.	Pioneered a precision antimicrobial therapy that transforms pathogenic bacteria into therapeutic targets and immune stimulants.

Maturity levels are defined as follows: Level 0, proof-of-concept stage; Level 1, preclinical studies; Level 2, preliminary clinical evidence available.

### 4.1. Diet

The overall dietary structure profoundly influences the composition and function of the gut microbiota, thereby modulating systemic immune status and responses to immunotherapy.^100^ Epidemiological evidence suggests that dietary factors may account for a relatively high proportion of CRC cases in Western populations. CRC is more likely to develop in individuals with diets low in unprocessed grains, dietary fiber, and vegetables and high in animal fats, red meat, and processed meats.^100^ A high-fat diet can significantly alter the gut microbiota composition, leading to the activation of carcinogenic pathways, alterations in the TIME, and DNA damage.^101^^,^^102^ A six-month intervention study targeting young Chinese individuals demonstrated a significantly reduction in gut microbiota diversity in the high-fat diet group. The relative abundance of the *Bacteroides* and *Alistipes* increased substantially, while that of *Faecalibacterium* decreased markedly in this cohort.^103^ A high-fat diet not only alters the composition of the gut microbiota but also profoundly affects microbial metabolic activities, resulting in increased production of harmful metabolites that contribute to colorectal carcinogenesis.^104^ In contrast, Mediterranean-style diets or high-fiber diets rich in dietary fiber and polyphenols help maintain a diverse gut ecosystem enriched with short-chain fatty acid-producing bacteria. Such dietary patterns enhance intestinal barrier integrity and reduce systemic inflammation levels, thereby creating a more favorable systemic and local microenvironment for immunotherapy.^100^ Furthermore, time-restricted feeding (TRF), as a dietary intervention that reinforces host and microbial circadian rhythms, can improve metabolic parameters and microbial composition, holding potential for enhancing therapeutic efficacy.^72^

It is important to note that not all dietary fibers have the same effect on the gut microbiota. Fermentable fibers, such as inulin, fructooligosaccharides (FOS), and resistant starch, serve as important dietary sources for the gut microbiota. They can be selectively utilized by specific gut microbiota (such as SCFA-producing bacteria), thereby promoting the growth and activity of these beneficial bacteria. In contrast, nonfermentable or low-fermentable fibers primarily function as bulk agents, promoting intestinal motility and stool formation, while exerting limited effects on microbiota composition or SCFA production.^105^^,^^106^ This distinction holds significant implications for immunotherapy. Strategies to enhance the efficacy of ICIs through dietary intervention should prioritize fermentable fiber that actively shapes the immunomodulatory metabolite pool, rather than nonfermentable fibers with limited metabolic impact. Future research should explore whether specific fiber types can be selected based on individual patient microbiome characteristics to optimize ICI response.

### 4.2. FMT

FMT is a biological intervention that treats disease by transferring functional microbial communities into patients, thereby restoring the normal composition and function of the gut microbiota. Originally developed for the treatment of Clostridioides difficile infection, FMT has been increasingly explored for therapeutic applications in inflammatory bowel disease, irritable bowel syndrome, and various cancers.^107^ Numerous studies have demonstrated that FMT can enhance the response of melanoma patients to immunotherapy.^108-110^ This has prompted researchers to expand their investigations into CRC, particularly focusing on CRC patients with MSS. Preclinical studies demonstrate that FMT enhances the therapeutic efficacy of immunotherapy in CRC through synergistic mechanisms, including remodeling of gut microbial composition, modulation of microbial gene functions, and altering in plasma metabolomic profiles.^111^ In patients with treatment-resistant MSS-type tumors, third-line therapy combining FMT (30 oral capsules daily for 3 consecutive days per 3-week cycle) with tislelizumab and fruquintinib achieved a disease control rate of up to 95% and significantly prolonged survival.^112^ Although FMT holds considerable promise for the treatment of CRC, there are still challenges, which we will discuss in detail in Section 5. Future large-scale randomized controlled trials (RCTs) are essential to validate its efficacy and to establish a robust foundation for more precise patient selection and optimization of treatment strategies.

### 4.3. Probiotics and specific prebiotics

Probiotics' ability to modulate the immune system and exhibit antitumor properties has gained increasing recognition. Probiotics can synergize with ICIs to suppress the initiation and progression of CRC, including *Lactobacillus rhamnosus*(*L. rhamnosus*),^113^^,^^114^ *C. butyricum,*^115^ and *Lactococcus lactis* (*L. lactis*).^116^

In recent years, prebiotics have increasingly been recognized as a promising adjunctive therapeutic approach for patients with CRC. Prebiotics are nondigestible dietary components that escape host digestion and are selectively utilized by beneficial gut microorganisms, thereby promoting their growth or activity and contributing to enhanced host health.^117^ The oral in situ forming retention-type inulin gel enhances colonic retention, enables in situ modulation of commensal microbiota, and thereby potentiates the antitumor efficacy of ICIs therapy. It exhibits a well-documented safety profile and is amenable to scalable for production.^118^ Pectin enhances the production of SCFAs by favorably modulating the gut microbiota, thereby improving the efficacy of immunotherapy in CRC.^119^ Ganoderma lucidum polysaccharides enhance T cell-mediated antitumor immunity and suppress CRC progression by modulating gut microbiota composition, increasing serum butyrate levels, and inhibiting inducible dopamine oxidase activity. Moreover, combination therapy with ICIs synergistically improves therapeutic in CRC treatment.^120^

Probiotics and prebiotics primarily enhance the antitumor effects of ICIs by modulating the composition of the gut microbiota, emerging as promising adjunctive strategies in cancer therapy. However, it is important to note that not all probiotics are beneficial in the context of immunotherapy. As discussed in more detail in Section 5.2, the effects of probiotics are highly strain-specific and context-dependent, and their therapeutic potential can be influenced by the patient's baseline microbiota composition. These complexities underscore the need for caution and individualized approaches when considering probiotic supplementation during ICI therapy. This is precisely the challenge that next-generation Live Biotherapeutic Products (LBPs), discussed below, aim to address through rigorous mechanistic and clinical development.

### 4.4. Use antibiotics judiciously

In contrast to strategies that broadly promote beneficial bacteria, targeted eradication aims to specifically eliminate particular pathogenic species that drive tumor progression and shape an immunosuppressive microenvironment. This represents a precision intervention philosophy shifting from “reinforcing the host” to “removing the pathogen”.

Clinical observations consistently indicate that the use of broad-spectrum antibiotics around the time of ICI therapy is significantly associated with poorer survival outcomes in patients with various cancers, including non-small-cell lung cancer and melanoma.^121^^,^^122^ The detrimental effect primarily stems from the nonselective depletion of immunostimulatory commensal bacteria, thereby undermining the foundation for immunotherapy.^123^ Research has found that microbial diversity is significantly correlated with the efficacy of ICI therapy, and excessive antibiotic use leads to low microbial diversity.^124^ These studies have spurred the development of precision antimicrobial strategies. For example, researchers have developed liposomal antibiotics designed to selectively deliver drugs to tumor-associated *F. nucleatum*. This not only kills the bacteria but also generates bacterial neoantigens that can induce potent, tumor-specific T cell responses, creating a synergistic “vaccine-like” effect with ICIs.^125^ This strategy ingeniously converts a pathogenic factor into both a therapeutic target and an immunological adjuvant. In the future, integrating cutting-edge technologies such as phage therapy and CRISPR-mediated precision antimicrobials holds promise for achieving microbiome editing with higher specificity and controllability.

## 5. Clinical evidence and future perspectives

To overcome resistance to CRC immunotherapy, combining ICIs with other treatment modalities is crucial. Strategies to modulate the microbiome are increasingly becoming an important component of these combination therapies.

### 5.1. Microbiome-based precision combination therapy: living biopharmaceuticals and targeted interventions

Early-phase trials of FMT primarily explored its safety and potential to reverse resistance in refractory patients who progressed after receiving ICI therapy. As previously described, the RENMIN-215 trial (NCT04729322) combined FMT with tislelizumab (anti-PD-1) and fluquintidine for treatment-refractory MSS mCRC, reporting encouraging objective response rates and confirming that clinical responses correlate with the transplantation of beneficial microorganisms and their metabolites from donors.^112^ Although CRC data is still being accumulated, pioneering studies in melanoma and other tumor types relatively sensitive to ICIs provide strong support for the mechanism. For example, a small study (NCT03341143) in patients with advanced melanoma resistant to anti-PD-1 therapy demonstrated that following FMT from an ICI responder, some patients regained sensitivity to PD-1 inhibitors and achieved tumor shrinkage. Analysis of the tumor immune microenvironment in responders revealed increased CD8^+^ T-cell infiltration, reduced immunosuppression, and a shift in gut microbiota composition toward donor-like beneficial bacterial profiles.^110^ These findings provide crucial proof-of-concept for implementing similar combination strategies in MSS-CRC.

Although FMT shows considerable promise for treating CRC, challenges remain. In FMT research, the “super-donor” hypothesis remains a key issue of significant interest that has yet to be fully resolved. This hypothesis suggests that fecal matter from certain donors can consistently produce significant clinical benefits, whereas that from other donors cannot. This phenomenon has prompted researchers to explore whether “super donors” with unique microbiome characteristics exist, whose feces may contain specific types of microorganisms or more robust microbial communities, thereby delivering sustained clinical benefits across a broader patient population.^126^ However, a reassessment of the evidence for “super donors” reveals that the existence of such donors has not yet been fully substantiated.^127^^,^^128^ The efficacy of FMT is not determined by a single factor but is influenced by multiple factors. The composition of the donor microbiota, the recipient's baseline gut microbiota, immune status, and the FMT protocol all affect its therapeutic outcomes.^129^ Although FMT demonstrates significant efficacy in treating certain diseases, its safety remains the primary challenge hindering widespread clinical application. Key risks include pathogen transmission, transfer of antibiotic resistance genes, and unknown long-term safety.^130^^,^^131^ To minimize the risks associated with FMT and enhance its safety, it is crucial to optimize FMT protocols, implement rigorous donor screening programs, and establish standardized fecal preparation procedures.^132^

Compared to FMT, using well-defined, clearly characterized live bacterial products or microbial metabolites for modulation offers superior advantages in quality control, safety, and reproducibility. VE800, a live bacterial product comprising 11 human-derived bacterial strains, demonstrated in preclinical studies that it activates CD8^+^ T cells and enhances the efficacy of ICIs. A Phase I clinical trial (NCT04208958) evaluated the safety and preliminary efficacy of VE800 in combination with nivolumab (anti-PD-1) in patients with advanced or metastatic solid tumors, including CRC. Preliminary data demonstrated favorable tolerability and observed changes in biomarkers of immune activation. Another product, MRx0518, is a live bacterium (*Enterococcus gallinarum*) with immune-stimulating properties. It is currently being studied in Phase I/II trials (NCT03637803) in combination with pembrolizumab for various solid tumors, including CRC.

However, the clinical translation of LBPs like VE800 faces significant production and delivery challenges. Many of these strains are strictly anaerobic bacteria that are highly sensitive to oxygen. They must be produced, stored, and formulated under strictly anaerobic conditions to maintain their viability. More challenging is the need to ensure these bacteria can withstand the harsh environment of the upper digestive tract—including gastric acid, bile salts, and digestive enzymes—and reach the colon in a functionally active state to exert their immunomodulatory effects.^133^ To overcome these obstacles, researchers have developed strategies such as polymer encapsulation to enhance anaerobic bacteria storage,^134^^,^^135^ employing enteric-coated capsules to protect bacterial survival during gastric transit,^136^ utilizing freeze-drying technology combined with lyophilization stabilizers to improve stability,^137^^,^^138^ and developing spore preparations with greater resistance to environmental impacts.^139^ In summary, the successful development and clinical translation of LBPs rely on advanced formulation technologies. These technologies effectively protect live bacteria from damage during production, storage, and exposure to the gastrointestinal environment, ensuring they reach the target site with high potency and consistent dosage to deliver the intended therapeutic effect.

### 5.2. Exploration and controversy surrounding traditional probiotics as an adjuvant therapy

Beyond the aforementioned invasive or highly targeted interventions, clinical exploration of oral probiotics as a more convenient adjunct therapy in combination with ICIs is ongoing, though the results are more complex.

Multiple preclinical studies suggest that specific probiotic combinations can enhance responses to ICIs in mouse models. Based on these findings, observational studies and small-scale interventional trials have begun exploring the use of probiotics in cancer patients. A Phase I clinical trial (NCT03829111) demonstrated that CBM588 in combination with nivolumab plus ipilimumab significantly improved progression-free survival (PFS) in patients with metastatic renal cell carcinoma (mRCC), with manageable safety.^140^

Despite encouraging preclinical data, recent clinical observations have raised concerns about the misuse of commercially available probiotics during cancer immunotherapy. In a cohort of melanoma patients receiving ICI therapy, those who self-reported using over-the-counter probiotics demonstrated poorer treatment responses, although the difference did not reach statistical significance.^141^ It is speculated that certain probiotic strains may inadvertently displace native beneficial microbiota, produce immunosuppressive metabolites, or fail to colonize the gut in cases of dysbiosis. These findings underscore the strain specificity and environmental dependence of probiotic effects, suggesting that caution is warranted. Therefore, patients are advised not to use unregulated probiotic products on their own during ICI therapy. Clinicians should await evidence from well-designed clinical trials before recommending specific probiotic formulations as adjuncts to immunotherapy. Determining the optimal microbial intervention strategies, identifying the most effective microbial “formulations”, defining predictive biomarkers, and understanding their underlying mechanisms are core challenges that future large-scale clinical studies must address.

### 5.3. FDA approvals and the evolving treatment landscape

Currently, the regulatory landscape for ICIs in CRC is primarily centered on the MSI-H/dMMR population. However, the scope of clinical approvals is progressively expanding toward earlier disease stages and novel combination therapeutic regimens.

While microbiota-targeted therapies have yet to receive formal regulatory approval in the CRC field, current regulatory trends and clinical development trajectories clearly indicate a fundamental paradigm shift. Traditional nonspecific probiotic interventions are being superseded by mechanistically defined LBPs, which are pursuing regulatory breakthroughs through innovative clinical trial designs. Prominent programs—such as Seres Therapeutics' SER-155 and Vedanta Biosciences' VE800—have established comprehensive drug development frameworks spanning strain identification, mechanism-of-action validation, and GMP-compliant manufacturing. Their clinical trials strictly adhere to the regulatory pathways mandated for biological products. Significantly, the FDA has not only established a distinct LBP classification for such candidates but has also demonstrated a proactive regulatory stance by granting multiple Fast Track designations, signaling growing regulatory attention and paving the way for future approvals in this emerging field. This evolution profoundly restructures the therapeutic landscape: microbiota signatures are transitioning from auxiliary biomarkers to core determinants driving clinical decision-making.

## 6. Conclusion

ICIs demonstrate considerable potential in the treatment of CRC, yet their clinical application remains constrained by challenges including a narrow patient population and drug resistance. The gut microbiota, as a key regulator of ICI efficacy, constructs a complex immunoregulatory network through specific bacterial strains, metabolites, and bioactive substances. This network can both enhance antitumor immune responses and potentially induce treatment resistance by inducing immunosuppression, with this regulatory function being influenced by circadian rhythms. Microbiome-targeting strategies such as dietary interventions, FMT, specific probiotic and prebiotic supplementation, and judicious use antibiotic offer viable approaches to overcoming treatment bottlenecks in ICI therapy. The synergistic effects of probiotics with ICIs have been supported by multiple studies, and FMT has also demonstrated potential for improving disease control rates in patients with refractory MSS CRC.

Research on the gut microbiota and its role in the treatment of CRC with ICIs has advanced significantly, however, several questions remain unanswered. Current research primarily focuses on individual bacterial strains or specific metabolites. When confronting complex microbial communities, the mechanisms underlying CRC development and the efficacy of ICIs remain poorly understood. Moreover, the relationship between the microbiota and circadian rhythms remains in its preliminary stages of research, with the underlying mechanisms of interaction yet to be fully elucidated. Clinical translation remains in its infancy, with intervention protocols lacking standardization and validation through large-scale RCTs.

Future efforts should focus on: (1) validating microbiota modulation strategies in large-scale randomized controlled trials to establish standardized protocols; (2) deciphering the complex interactions within microbial communities rather than single species; (3) elucidating the intricate crosstalk between gut microbiota, host circadian rhythms, and immunity to develop chrono-therapeutic approaches; and (4) moving towards personalized microbiome-based interventions to expand the beneficiary population of ICI therapy in CRC.

## Data Availability

Not applicable.
